# Forebrain Cholinergic Dysfunction and Systemic and Brain Inflammation in Murine Sepsis Survivors

**DOI:** 10.3389/fimmu.2017.01673

**Published:** 2017-12-15

**Authors:** Nahla Zaghloul, Meghan E. Addorisio, Harold A. Silverman, Hardik L. Patel, Sergio I. Valdés-Ferrer, Kamesh R. Ayasolla, Kurt R. Lehner, Peder S. Olofsson, Mansoor Nasim, Christine N. Metz, Ping Wang, Mohamed Ahmed, Sangeeta S. Chavan, Betty Diamond, Kevin J. Tracey, Valentin A. Pavlov

**Affiliations:** ^1^Cohen Children’s Medical Center, Northwell Health, New Hyde Park, NY, United States; ^2^Neonatology Research Laboratory, Feinstein Institute for Medical Research, Northwell Health, Manhasset, NY, United States; ^3^Center for Biomedical Science, Feinstein Institute for Medical Research, Northwell Health, Manhasset, NY, United States; ^4^Donald and Barbara Zucker School of Medicine at Hofstra/Northwell, NY, United States; ^5^Laboratory of Neurobiology of Systemic Illness, Department of Neurology, Instituto Nacional de Ciencias Médicas y Nutrición Salvador Zubirán, Mexico City, Mexico; ^6^Laboratory of Neurobiology of Systemic Illness, Department of Infectious Diseases, Instituto Nacional de Ciencias Médicas y Nutrición Salvador Zubirán, Mexico City, Mexico; ^7^Neuropathology-Anatomic Pathology, Northwell Health, New Hyde Park, NY, United States; ^8^Center for Immunology and Inflammation, Feinstein Institute for Medical Research, Northwell Health, Manhasset, NY, United States; ^9^Center for Bioelectronic Medicine, Feinstein Institute for Medical Research, Northwell Health, Manhasset, NY, United States; ^10^Center for Autoimmune and Musculoskeletal Diseases, The Feinstein Institute for Medical Research, Manhasset, NY, United States

**Keywords:** sepsis, sepsis survival, cytokines, inflammation, brain cholinergic system, neuroinflammation

## Abstract

Sepsis, a complex disorder characterized by immune, metabolic, and neurological dysregulation, is the number one killer in the intensive care unit. Mortality remains alarmingly high even in among *sepsis survivors* discharged from the hospital. There is no clear strategy for managing this lethal chronic sepsis illness, which is associated with severe functional disabilities and cognitive deterioration. Providing insight into the underlying pathophysiology is desperately needed to direct new therapeutic approaches. Previous studies have shown that brain cholinergic signaling importantly regulates cognition and inflammation. Here, we studied the relationship between peripheral immunometabolic alterations and brain cholinergic and inflammatory states in mouse survivors of cecal ligation and puncture (CLP)-induced sepsis. Within 6 days, CLP resulted in 50% mortality vs. 100% survival in sham-operated controls. As compared to sham controls, sepsis survivors had significantly lower body weight, higher serum TNF, interleukin (IL)-1β, IL-6, CXCL1, IL-10, and HMGB1 levels, a lower TNF response to LPS challenge, and lower serum insulin, leptin, and plasminogen activator inhibitor-1 levels on day 14. In the basal forebrain of mouse sepsis survivors, the number of cholinergic [choline acetyltransferase (ChAT)-positive] neurons was significantly reduced. In the hippocampus and the cortex of mouse sepsis survivors, the activity of acetylcholinesterase (AChE), the enzyme that degrades acetylcholine, as well as the expression of its encoding gene were significantly increased. In addition, the expression of the gene encoding the M1 muscarinic acetylcholine receptor was decreased in the hippocampus. In parallel with these forebrain cholinergic alterations, microglial activation (in the cortex) and increased *Il1b* and *Il6* gene expression (in the cortex), and *Il1b* gene expression (in the hippocampus) were observed in mouse sepsis survivors. Furthermore, microglial activation was linked to decreased cortical ChAT protein expression and increased AChE activity. These results reinforce the notion of *persistent inflammation-immunosuppression and catabolic syndrome* in sepsis survivors and characterize a previously unrecognized relationship between forebrain cholinergic dysfunction and neuroinflammation in sepsis survivors. This insight is of interest for new therapeutic approaches that focus on brain cholinergic signaling for patients with chronic sepsis illness, a problem with no specific treatment.

## Introduction

Sepsis is a clinical syndrome manifested by a spectrum of immune, metabolic, and neurological derangements. Although sepsis remains the most frequent cause of death in intensive care units, advances in critical care medicine have substantially reduced sepsis mortality during hospitalization (1, 2). However, sepsis continues to kill for a long time after patients are discharged from the hospital (3–6). Many *sepsis survivors* are unable to return to independent living and develop chronic illness, characterized by severe functional disabilities, which ultimately result in death (4–8).

The brain is severely affected in sepsis. In acute settings a frequent event is sepsis-associated encephalopathy, which ranges from confusion and delirium to coma (9). Among other derangements, brain neurotransmission dysregulation, microglial activation, and proinflammatory signaling have been described within the spectrum of this characteristic encephalopathy (9–11). This neurological complication is an independent predictor of sepsis mortality (9). Persistent long-term cognitive impairment has been also reported in survivors of sepsis and other critical illnesses (3, 4, 12). This cognitive impairment can be as severe as the one observed in moderate traumatic brain injury and mild Alzheimer disease (13). Pathophysiological events in the brain underlying cognitive impairment in the context of other functional disabilities associated with metabolic and immune dysfunction remain poorly understood. Recently, the condition of *persistent inflammation-immunosuppression and catabolic syndrome* was described in sepsis survivors, who develop debilitating and lethal chronic illness (14, 15). Peripheral inflammation and increased circulating cytokine levels have been linked to brain proinflammatory signaling and altered brain function (16, 17). In a murine model, systemic inflammation during endotoxemia was indicated as a trigger of profound glial activation and apoptotic neuronal death in the hippocampus (18). Increased brain cytokine and chemokine transcription with microglial activation and decreased neuronal cell density in the cortex and hippocampus of endotoxemic rats was also reported (19). Cecal ligation and puncture (CLP)-induced sepsis in mice results in impaired learning and memory, along with chronic loss of dendrites in the hippocampus (20, 21). In murine sepsis survivors, proinflammatory signaling induced by HMGB1 mediates cognitive deterioration (21). A recent study also reported long-term brain alterations in mouse sepsis survivors, including forms of cognitive deficit and neuroinflammation (22).

Cognition, including attention, learning, and memory, is regulated by brain cholinergic signaling. Cholinergic neurons in the basal forebrain constitute a major neuromodulatory system with a critical role in this regulation (23, 24). Neurodegeneration of basal forebrain cholinergic neurons is implicated in cognitive impairment in Alzheimer’s disease (23). These neurons also regulate neuroplasticity allowing functional recovery following brain injury (25). Recent findings have also characterized a role for brain cholinergic signaling in controlling peripheral immune responses and inflammation (26–32). Despite the important involvement of the brain cholinergic system in cognition and the neural control of inflammation, the understanding of its function in the context of sepsis is limited. We recently reported altered gene expression of brain cholinergic system markers in parallel with increased circulating cytokine levels and inflammation in the brain (neuroinflammation) of endotoxemic mice (33). Reduced cholinergic innervations of cortical areas and neuronal loss in the hippocampus and long-term cognitive impairment, similar to that observed in Alzheimer’s disease, was reported in rats that survived endotoxemia (34). Here, we studied the relationship between peripheral immune and metabolic alterations and forebrain cholinergic signaling and neuroinflammation in murine survivors of CLP-induced sepsis.

## Materials and Methods

### Animals

Male BALB/c mice (24–28 g, Taconic) were used in experiments. Animals were allowed to acclimate for at least 2 weeks prior to initiating the experiment. All animals were housed in standard conditions (room temperature 22°C with a 12 h light–dark cycle) with access to regular chow and water. All animal experiments were performed in accordance with the National Institutes of Health Guidelines under protocols approved by the Institutional Animal Care and Use Committee and the Institutional Biosafety Committee of the Feinstein Institute for Medical Research, Northwell Health, Manhasset, NY, USA.

### Cecal Ligation and Puncture

A standardized model of CLP-induced severe polymicrobial sepsis was used, as previously described (31, 35, 36). Mice were anesthetized using ketamine 100 mg/kg and xylazine 8 mg/kg, administered intramuscularly. Abdominal access was gained *via* a midline incision. The cecum was isolated and ligated with a 6-0 silk ligature below the ileocecal valve and then punctured once with a 22 G needle. Stool (approximately 1 mm) was extruded from the hole, and the cecum was placed back into the abdominal cavity. The abdomen was closed with two layers of 6-0 Ethilon sutures. An antibiotic, Primaxin (Imipenem-Cilastatin, 0.5 mg/kg, subcutaneously, in a total volume of 0.5 ml/mouse) was administered immediately after CLP as part of the resuscitation fluid. Mice were monitored for survival and sepsis-associated clinical signs twice daily for the first 7 days, and then daily for the remaining of 14 days. Sham-operated animals had the cecum isolated and then returned to the peritoneal cavity without being ligated or punctured. Sham animals also received antibiotics and resuscitative fluid as described above.

### Brain Tissue Preparation

After collecting blood for cytokine analysis, brains were isolated on ice. For brain immunostaining, brain isolation was preceded by transcardial perfusion with saline and 4% paraformaldehyde. For acetylcholinesterase (AChE) activity determination and gene expression by qPCR, the cerebral cortices and the hippocampi were dissected on ice using a binocular dissection microscope. Brain tissue was snap frozen on dry ice and transferred to storage at −80°C.

### Serum Cytokine and Metabolic Molecule Determination

Blood was collected immediately after euthanasia by cardiac puncture. To obtain serum samples, blood was allowed to clot for 1.5 h and centrifuged at 5,000 rpm (1,500 *g*) for 10 min. Supernatants (sera) were collected and stored at −20°C until cytokine analyses. Interleukin (IL)-6, IL-1β, chemokine (C-X-C motif) ligand (CXCL1), IL-12p70, IL-5, IL-10, and TNF were determined by using the V-PLEX proinflammatory panel 1 mouse kit (Meso Scale Discovery, Gaithersburg, MD, USA), according to manufacturer’s recommendations. HMGB1 was determined by western blot as previously described by Yang et al. (37). Insulin, leptin, plasminogen activator inhibitor-1 (PAI-1) (total), and resistin were measured by using milliplex map mouse adipokine magnetic bead panel—endocrine multiplex assay (Millipore, USA) according to manufacturer’s recommendations.

### Brain Sample Isolation and qPCR Analysis

Fresh brains were collected in RNA later (Ambion^®^) 2 weeks following CLP. RNA isolation from cortex and hippocampus was performed using RNeasy Plus mini kit (Qiagen, Germantown, MD, USA) after tissue homogenization with the Bullet Blender Homogenizer (Next Advance, Averill Park, NY, USA) and the recommended bead lysis kit. Because of limited RNA levels found in the hippocampus, three tissue samples were combined from the same treatment groups before RNA extraction. RNA concentrations were measured using NanoDrop 1000 (Thermo Fisher Scientific Inc., Waltham, MA, USA). Specific primers for mouse *Il1b, Il6, Ache, Chat, Chrm1*, and *Gapdh* (internal reference gene) were designed using the Universal Probe Library Assay Design Center (Roche Applied Sciences, Indianapolis, IN, USA) with the assigned probes as listed in Table 1. RNA (100 ng/reaction) was amplified using a Eurogentec reverse-transcriptase qPCR master mix (AnaSpec, EGT Corporate Headquarters, Fremont, CA, USA) on a LightCycler 480 (F. Hoffmann-La Roche Ltd., Basel, Switzerland), and the data were analyzed by using the Roche LightCycler 480 SW 1.5 software. Relative changes in mRNA expression were calculated as fold-changes (normalized using *Gapdh*) by using the comparative Ct (ΔΔCt) method (38).

**Table 1:** Probes used in qPCR analyses.

Primer	Probe
*Il1b*	Left AGTTG ACGGACCCCAAAAG Right AGCTG GATGCTCTCATCAGG
*Il6*	Left GCTACCAAACTGGATATAAT CAGGA Right CCAGGTAGCTATGGT ACTCCAGAA
*Ache*	Left TTAGGGCTGGGATATAATACGAC Right GCCCCTAGTGGGAGGAAGT
*Chat*	Left AAGCTTCCACGCCACTTTC Right AGAGCCTCCGACGAA GTTG
*Chrm1*	Left GGTCCCAGGAGACACTGC Right TCAGAGTAAGGGCATCACCA
*Gapdh*	Left GAGCCCGCAGCCTCCCGCTT Right CCCGCGGCCATCACGCCACAG

### Acetylcholinestrase (AChE) Activity Assay

Brain tissue homogenates from cortex and hippocampus were prepared in a 20 mM Tris-HCl buffer (pH7.3), containing 10 mM MgCl_2_, 50 mM NaCl, a protease inhibitor and zirconium beads using a bullet-blender homogenizer (Next Advance, Troy, NY, USA), according to the manufacturer’s recommendations. Homogenates were centrifuged (24,500 *g*) and the pellets were resuspended in equal volumes of 0.1 M NaHPO_4_ buffer (pH 7.4–8.0) and 0.1% Triton-X-100. Following centrifugation (24,500 *g*) the resultant supernatants (containing membrane bound cholinesterase) were used an AChE activity assay based on the widely used Ellman’s procedure (39) and its modification (40). A butyrylcholinesterase inhibitor was added to the reaction mix. The reaction was initiated by adding acetyl thiocholine substrate, a thiolester. The amount of thiocholine formed reflects AChE activity. The color of the reaction mix was read at 412 nm. Calculations were performed using molar absorptivity (*ε*) for thionitrobenzoate at 412 nm. The results are expressed as millimoles of thiocholine released per minute at 25°C per 1 ml of lysate per 1 mg of protein.

### Immunohistochemical Staining

Following euthanasia by CO_2_ asphyxiation, animals were perfused transcardially with saline, and then 4% paraformaldehyde. Whole brain tissue was fixed in 4% paraformaldehyde for 24 h, and then processed, paraffin embedded, and sectioned sagittally at 6 µm thickness using a microtome (Leica Biosystems). The Paxinos and Franklin mouse brain atlas (41) was used to establish laterality (on the coronal plane) for serial sections to include areas of interest. For immunofluoresence, following deparaffinization and antigen retrieval, sections were incubated for 2 h at room temperature in TBS + 1% Triton-X + 10% donkey serum. Sections were incubated for 24 h at 4°C with primary antibodies, followed by 2 h incubation at room temperature with the appropriate secondary antibody with DAPI. All images were captured on a confocal microscope (Olympus Fluoview 300 Confocal Microscope). The following primary antibodies were used: anticholine acetyltransferase (anti-ChAT) antibody [Millipore (1:100) Billerica, MA, USA] and Iba-1 [Wako (1:400), Richmond, VA, USA]. The secondary antibodies which were used include Cy3 donkey antigoat IgG Cy3 and donkey antirabbit IgG Alexa Fluor 488 (Jackson Immunoresearch, 1:125, West Grove, PA, USA). For negative control, sections were incubated with TBS + 1% Triton-X + 10% donkey serum for 24 h (no primary antibody added) followed by 2 h incubation with the appropriate secondary antibody with DAPI.

### Immunostaining Analysis

Digital images were obtained using Confocal software and were exported to Image J. Excitation and acquisition parameters were adjusted to fully eliminate pixel saturation and all images were collected under identical settings. Cholinergic (ChAT-positive neurons) were identified in the basal forebrain on the sagittal plane by referring to Allen Institute Allen Brain Atlas (http://mouse.brain-map.org). Cell counting (of ChAT positive neurons) was performed on four sections per animal (750 × 750 μm each) and four animals per group by an observer blinded to the experimental group.

### Cortical Neuron Cultures and LPS Microglial Activation

Cortical neuronal cultures were prepared from BALB/c mouse pups, as previously described (42) with some modifications. Briefly, postnatal day 0 newborn pup brains were harvested for neuronal cultures. Cortical gray matter was dissected, plated separately in six-well plates at 1 × 10^6^ per well (previously coated with laminin and poly-l-Ornithine, Sigma, St. Louis, MO, USA). Cultures were fed with neurobasal medium with B-27 supplements (Thermofisher, Waltham, MA, USA) and incubated at 37°C with 5% CO_2._ Seven-day-old neuronal cultures were treated for 16 h with 0.5 ml per well of microglial conditioned medium. Microglial conditioned media was obtained by incubating mouse microglia BV2 cells (ATCC CRL-2469) with LPS (10 µg/ml) in a humidified incubator (5% CO_2_, 37°C and 95% air) for 24 h.

### Choline Acetyl Transferase Protein Determination

Protein lysate was extracted from mouse primary cortical neuronal cell culture. Protein concentration was estimated using the Modified Lowry Protein Assay (Thermo Fisher Scientific, Rockford, IL, USA). Standard SDS-PAGE techniques were followed. After electrophoresis, proteins were transferred to a PVDF membrane using a Wet/Tank Blotting System (Bio-Rad, Hercules, CA, USA). Membranes were briefly washed, incubated with specific primary antibodies: goat anticholine acetyl transferase (ChAT; 1:1,000 Millipore, Billerica, MA, USA) or rabbit antiactin (1:1,000; Abcam, Cambridge, MA, USA) in 5% BSA with PBST overnight. After washing, the membranes were incubated with anti-goat HRP and anti-rabbit secondary antibodies (1:10,000) for 60 min, washed, processed using Amersham ECL detection systems (GE Healthcare, Piscataway, NJ, USA) and exposed to 8 × 10 Fuji X-Ray Film. Lysate from rat forebrain was used as positive control. Densitometric analysis was performed using Image J software.

### Statistical Analysis

All statistical tests were performed with Graph Pad Prism 6 software. Values are presented as mean ± SEM. Statistical analysis of mean differences between groups was performed by unpaired two-tailed Student’s *t*-test. All *P*-values and *n* values are indicated in figure legends and in the text. *P*-values ≤0.05 were considered significant.

## Results

### Immune and Metabolic States Are Altered in Murine Sepsis Survivors

**Figure 1:**
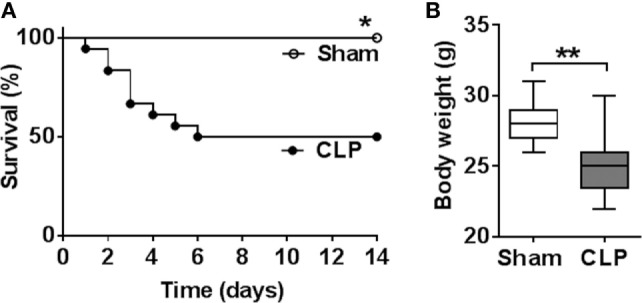
Sepsis mortality following cecal ligation and puncture (CLP) surgery and weight loss in sepsis survivors. **(A)** CLP causes mortality rate of 50% by day 6 and no later mortality until day 14 following surgery. *P = 0.0142, Log-rank test, *n* = 9 per group. **(B)** Fourteen days following CLP, mice exhibit lower body weight as compared to sham-operated controls. ***P = 0.0052, Student’s *t*-test, *n* = 9 per group.

**Figure 2:**
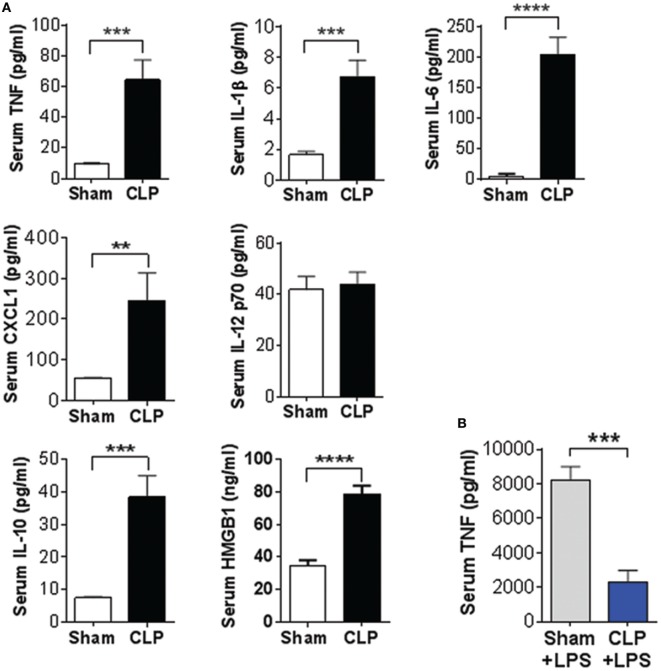
Cecal ligation and puncture (CLP)-sepsis alters circulating cytokine levels and suppresses LPS-induced immune responsiveness. **(A)** Increased serum cytokine levels in mouse sepsis survivors 14 days after CLP or sham surgery. **P = 0.0092, ***P = 0.0004 (TNF), ***P = 0.0002 (IL-1b, IL-10), ****P = 0.0001, Student’s *t*-test, *n* = 7–9 per group. **(B)** Sepsis survivors exhibit reduced LPS-induced serum TNF levels during endotoxemia when compared to sham controls. ***P = 0.001, Student’s *t*-test, *n* = 8–9 per group.

**Figure 3:**
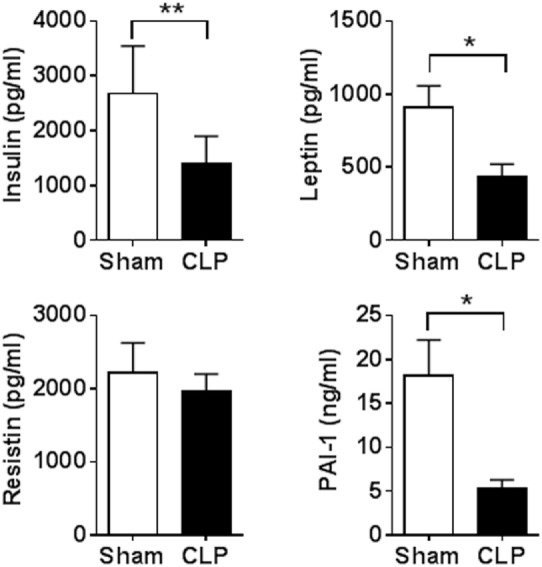
Cecal ligation and puncture (CLP)-sepsis alters metabolic markers. Serum levels of insulin, leptin, and plasminogen activator inhibitor-1 (PAI-1) were decreased in mouse sepsis survivors. *P = 0.0157 (leptin), *P = 0.0109 (PAI-1), **P = 0.0023, Student’s *t*-test, *n* = 8–9 per group.

First, we examined indices of the peripheral immune state and some metabolic markers in sepsis survivors. Two sets of mice with equal body weights were subjected to either CLP or sham surgery and survival was monitored for 14 days. A mortality rate of 50% was observed by day 6 with no further mortality following CLP (*n* = 9 per group) (Figure 1A). No mortality was observed in mice subjected to sham surgeries. The body weight of sepsis survivors was significantly lower when compared with sham controls 14 days postsurgery (*n* = 9 per group) (Figure 1B). Peripheral inflammation was examined in sepsis survivors and sham controls by determining serum cytokines levels. Fourteen days after the onset of disease, serum levels of TNF (*P* = 0.0004), IL-1β (*P* = 0.0002), IL-6 (*P* < 0.0001), CXCL1 (*P* = 0.0091), IL-10 (*P* = 0.0002), and HMGB1 (*P* = 0.0001) were significantly higher in CLP mice when compared with sham controls (*n* = 8–9 per group) (Figure 2A). No difference in serum IL-12p70 between the groups was observed (Figure 2A). In addition, very low IL-5 levels were determined in sham-operated controls and these levels were even lower in CLP mice (1.64 ± 0.19 vs. 0.87 ± 0.08 pg/ml, *P* = 0.027). To further characterize the impact of CLP on innate immune responsiveness, LPS (1 mg/kg, i.p.) was administered to sepsis survivors and sham mice 14 days after CLP- or sham-surgery and animals were euthanized 1.5 h later. As shown in Figure 2B endotoxin-induced serum TNF levels in CLP mice were fourfold lower (*P* = 0.001) than levels found in the sham controls (*n* = 8–9 per group). In addition to the inflammatory state, circulating levels of metabolic indices were altered in sepsis survivors. Circulating insulin (*P* = 0.0023), leptin (*P* = 0.0157), and PAI-1 (*P* = 0.0109) levels were significantly lower in CLP-survivors when compared to sham controls (*n* = 8–9 per group) (Figure 3). No difference in serum resistin levels between the groups was observed (Figure 3). Together these results indicate the presence of peripheral immune and metabolic dysregulation in mouse sepsis survivors.

### Forebrain Cholinergic Transmission Is Altered in Mice Sepsis Survivors

**Figure 4:**
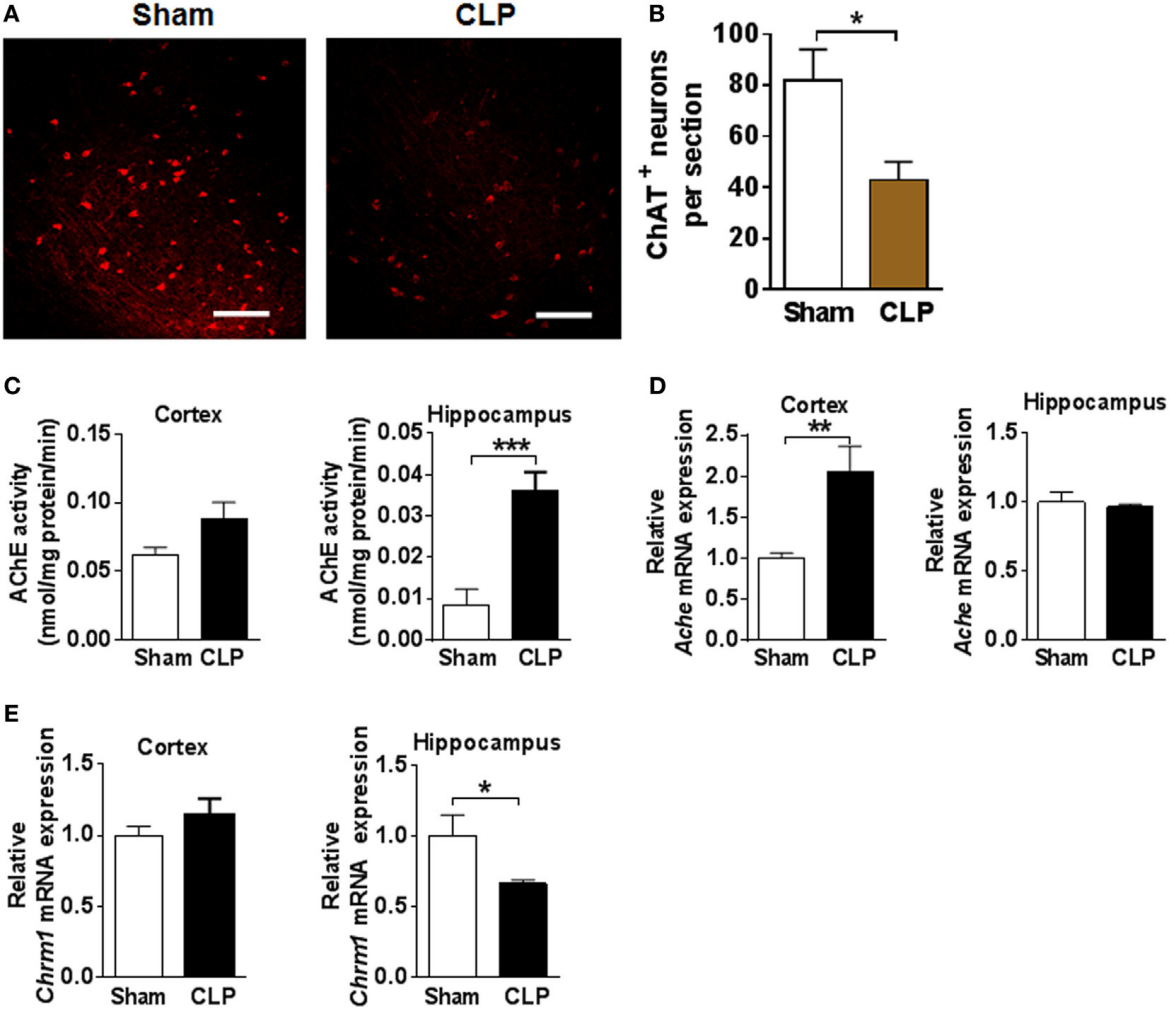
Cecal ligation and puncture (CLP) has a significant impact on basal forebrain cholinergic balance in sepsis survivors. **(A)** Representative images of choline acetyltransferase (ChAT) immunostaining in the basal forebrain of sham and CLP mice (scale bar = 100 μm). **(B)** Quantitative analysis of ChAT immunostaining in the basal forebrain of sham and CLP mice. *P = 0.0229, Student’s *t*-test, *n* = 5 per group. **(C)** Acetylcholinesterase (AChE) activity in cortex and hippocampus of sham and CLP mice. ***P = 0.0004, Student’s *t*-test, *n* = 8 per group. **(D)** Relative *Ache* mRNA expression in cortex and hippocampus of sham and CLP mice. **P = 0.0052, Student’s *t*-test, *n* = 8 per group. **(E)** Relative *Chrm1* mRNA expression in cortex and hippocampus of sham and CLP mice. *P = 0.0481, Student’s *t*-test, *n* = 8 per group.

To study the impact of CLP on the forebrain cholinergic transmission in sepsis survivors, we examined cholinergic neuronal bodies (somata) in the basal forebrain and molecular cholinergic components in two major projection areas of these neurons—the cortex and the hippocampus. ChAT, the enzyme responsible for the biosynthesis of acetylcholine, is a widely used marker for identifying cholinergic neurons. ChAT immunostaining revealed that the number of ChAT-positive neurons in the basal forebrain was significantly reduced in sepsis survivors compared to sham controls (*n* = 5 per group) (Figures 4A,B). ChAT colocalization with DAPI (Figure S1 in Supplementary Material) was used to precisely quantify ChAT-positive somata. Acetylcholine, released from cholinergic neurons, is rapidly degraded by AChE in the synaptic cleft between cholinergic and cholinoceptive neurons (23). AChE activity in the hippocampus was significantly increased among sepsis survivors (*n* = 8 per group) (Figure 4C). In addition, upregulation of *Ache* mRNA expression in the cortex was observed (*n* = 8 per group) (Figure 4D). Acetylcholine receptors on postsynaptic cholinoceptive neurons mediate cholinergic neurotransmission in the synaptic cleft. The M1 mAChR is predominantly postsynaptic, with a major role in processing cholinergic transmission in the cortex and hippocampus (43, 44). The expression of the gene encoding the M1 mAChR receptor, *Chrm1* was decreased in the hippocampus of mouse sepsis survivors (*n* = 8 per group) (Figure 4E). Comparing *Ache* and *Chrm1* gene expression in the hippocampus and the cortex of sham control mice showed lower *Ache* and higher *Chrm1* mRNA expression in the hippocampus (Figure S2 in Supplementary Material). These observations highlight dysfunctional forebrain cholinergic signaling in the sepsis survivors.

### Brain Inflammatory Indices Are Upregulated in Murine Sepsis Survivors

**Figure 5:**
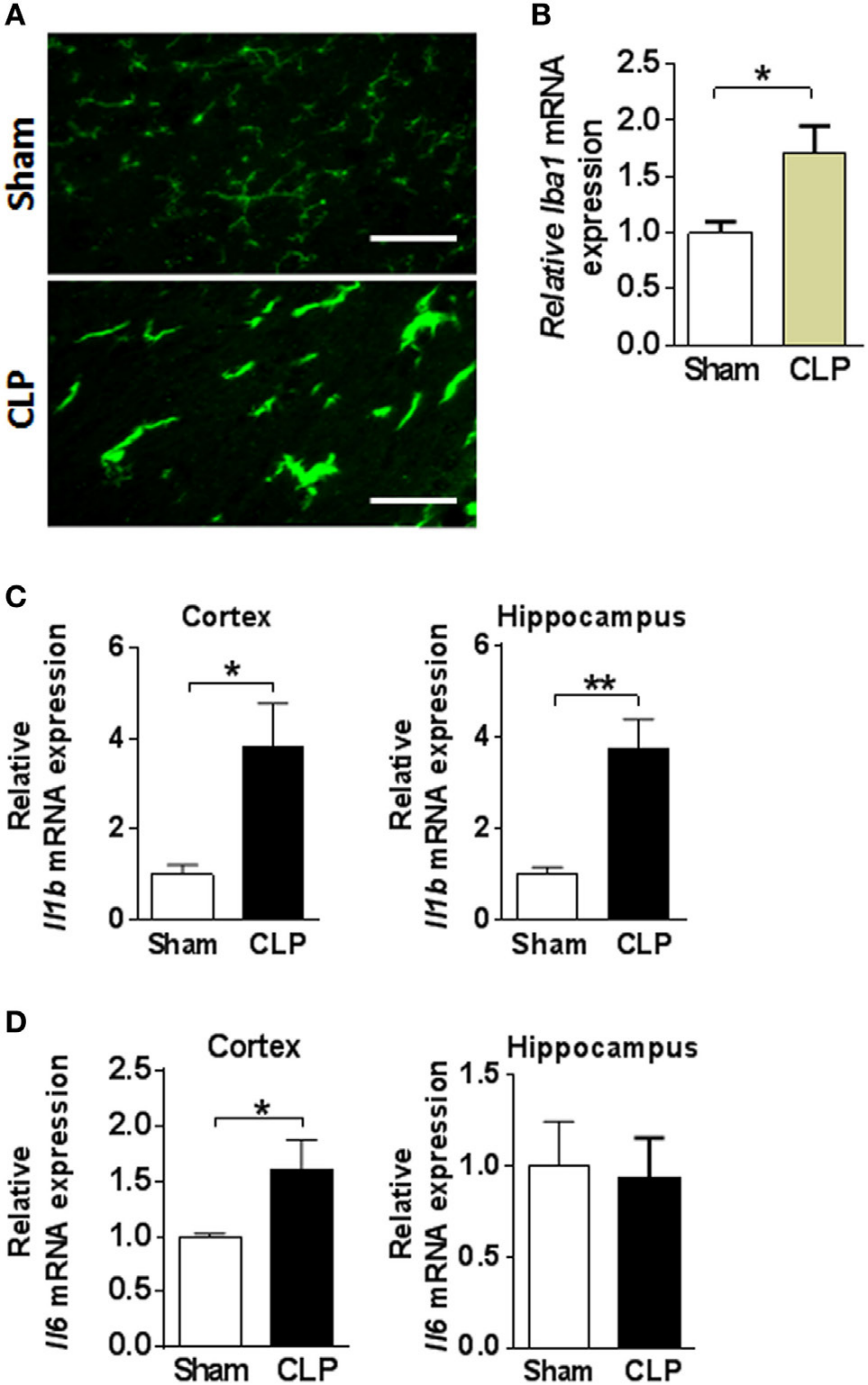
Cecal ligation and puncture (CLP) results in neuroinflammation in mouse sepsis survivors. **(A)** Representative images of Iba1immunostaining in cortex of sham and CLP mice (scale bar = 50 μm). **(B)** *Iba1* mRNA expression in cortex and hippocampus of sham and CLP mice. *P = 0.0139, Student’s *t*-test, *n* = 8 per group. **(C)** *Il1b* mRNA expression in cortex and hippocampus of sham and CLP mice. *P = 0.0123, **P = 0.0011, Student’s *t*-test, *n* = 8 per group. **(D)** *Il6* mRNA expression *P = 0.0455, Student’s *t*-test, *n* = 8 per group.

Next, we investigated inflammation in the brains of sepsis survivors by examining microglial activation and cytokine gene expression. Microglia, a major cell type with immune function in the brain mediates protective responses, but sustained microglial activation can have a deleterious impact on neuronal function (45, 46). Iba1 immunohistochemistry revealed morphological differences in microglia in the cortex between sepsis survivors and sham controls. Microglia in the cortex of sham operated controls exhibited typical ramified morphology, characterized by relatively small bodies and long, fine processes (*n* = 8 per group) (Figure 5A). In contrast, microglial hypertrophic cell bodies, and shortening and thickening of processes, indicative of activated microglia, were observed in the cortex of CLP mice (Figure 5A). In addition, a significant upregulation of *Iba1* gene expression was determined in the cortex of sepsis survivors when compared to sham controls (*n* = 8 per group) (Figure 5B). Microglial activation is associated with initiation of transcriptional pathways that lead to the release of cytokines and other inflammatory molecules (45). Accordingly, we found significantly upregulated *Il1b* gene expression in the cortex and hippocampus (*n* = 8 per group) (Figure 5C) and *Il6* gene expression in the cortex of mice following CLP (*n* = 8 per group) (Figure 5D). Astrocytes are another cell type in the brain that exhibit immune functions and astrogliosis is a distinct feature of neuroinflammation. Examination of astrocyte morphology using GFAP immunostaining revealed no gross differences between sepsis survivors and sham controls (data not shown). Collectively, these observations highlight the presence of sepsis-associated neuroinflammation with microgial activation and upregulated proinflammatory gene expression in the cortex and hippocampus of mice 14 days after CLP.

### Microglial Activation Affects Cholinergic System Components in Primary Cortical Neurons

**Figure 6:**
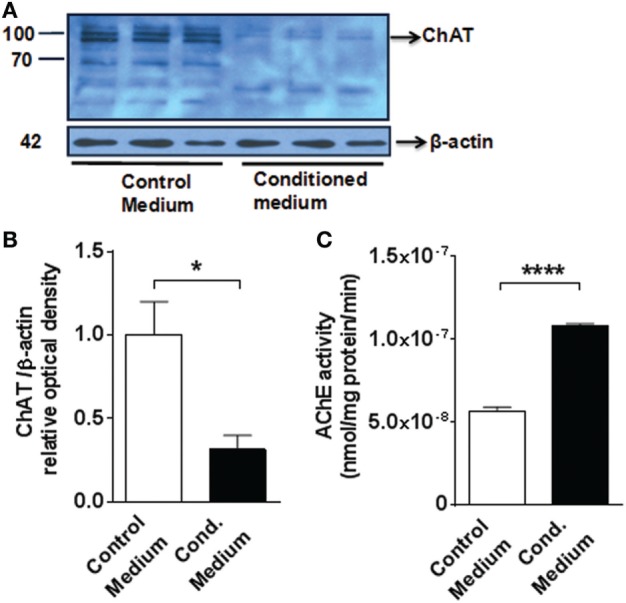
Medium from LPS-activated microglial cells decreases choline acetyltransferase (ChAT) protein expression and increases acetylcholinesterase (AChE) activity in mouse primary cortical neurons **(A)** western blot (MW 83 kDa) of ChAT of 7-day-old primary neuronal culture exposed to control or conditioned media (obtained from LPS-activated microglial BV2 cells). See Section “Materials and Methods” for details. **(B)** Fold change of the western blot determined using Image J to measure band intensities of ChAT normalized to β-actin.*P = 0.0343, Student’s *t*-test, *n* = 3 cultures per group, containing neurons obtained from five to six neonate brains each. **(C)** AChE activity in 7-day-old primary neuronal culture exposed to control or conditioned (Cond.) media ****P < 0.0001, Student’s *t*-test, *n* = 3 cultures per group, containing neurons obtained from five to six neonate brains each. The Student’s *t*-test was performed assuming normality and equal distribution of variance between the groups.

The relationship between microglial activation and the alterations in forebrain cholinergic signaling was further investigated by examining the impact of microglial activation on ChAT protein expression and AChE activity in primary cortical neurons. BV2 cells, a widely used murine microglial cell line (47), were incubated with 10 µg/ml LPS for 24 h and then the conditioned culture media was added to primary mouse cortical neurons for 16 h. In control experiments, basal conditioned BV2 culture media (in the absence of LPS) was added to primary mouse cortical neurons. Conditioned medium from LPS-activated microglial cells significantly decreased neuronal ChAT protein expression, as compared to control media treatment (microglial conditioned media in the absence of LPS) (*P* = 0.0343) (*n* = 3 per group) (Figures 6A,B). In addition, this treatment caused a significant increase in neuronal AChE activity in primary cortical neurons (*P* < 0.0001) (*n* = 3 per group) (Figure 6C). Together these observations suggest a causative role for activated microglia in altering cholinergic system components in cortical neurons.

## Discussion

Here, we show that mice surviving CLP-induced sepsis exhibit impaired immune and metabolic homeostasis, dysregulated forebrain cholinergic signaling, and neuroinflammation. In addition, our observations suggest that microglial activation, accompanying sepsis, can be linked to impaired brain cortical cholinergic signaling. These findings provide new insight into chronic sepsis illness and suggest potential therapeutic targets.

A growing number of patients surviving sepsis develop debilitating and lethal chronic illness. Recent data from two clinical trials revealed that the mortality of sepsis survivors (capable of independent living prior to severe sepsis) is approximately 30% within the first 6 months after the acute septic phase and the quality of life of one-third of the surviving patients is severely affected as indicated by their inability to return to independent living within 6 months (7). The increased risk of death among sepsis survivors persists for up to 5 years following the initial septic event, even after accounting for their underlying medical comorbidities (48). A recent study found that more than one fifth of patients surviving acute sepsis died within the first 2 years of causes not related to their health status before sepsis (5). Despite these staggering findings, there is no clear understanding of the pathophysiological events underlying the long-term sequelae of sepsis, including cognitive deterioration as a prerequisite for designing adequate treatments.

Here, we found increased serum IL-6, CXCL1, and HMGB1 levels in sepsis survivors 2 weeks post-CLP when compared to sham controls. Our current data describing dysregulated immune homeostasis in mouse sepsis survivors are in agreement with previous reports (49). In addition, higher serum IL-1β, and TNF and IL-10 levels were observed in the CLP survivors. The effect of CLP-sepsis on host innate immune responsiveness was investigated by administering endotoxin to sham mice and CLP survivors. CLP survivor mice showed significantly suppressed serum TNF levels following LPS challenge when compared to sham mice. These findings indicate the existence of a chronic inflammatory state in sepsis survivors, which coexists with suppressed innate immune function. This state, which we term *innate immune exhaustion* in sepsis survivors, may be a result of prolonged innate immune activation, which eventually leads to the inability of immune cells to adequately produce cytokines in response to immune challenges. Accordingly, early interventions directed toward neutralizing excessive innate immune activation may prevent or minimize immune suppression in sepsis survivors.

Metabolic dysregulation is a characteristic feature of sepsis pathobiology. Altered circulating levels of insulin, leptin, resistin, and PAI-1 have been previously indicated in acute settings of sepsis and these molecules have been implicated in sepsis pathogenesis (50, 51). However, no information is available about possible alterations in these metabolic molecules in sepsis survivors. Here, we found levels of insulin and leptin, a hormone and an adipokine associated with metabolic (predominantly anabolic) and immune functions (52) were lower in sepsis survivors. These results correlated with the lower body weight found in mouse sepsis survivors. We did not find a difference between the levels of the adipokine resistin in CLP survivors and sham controls. Levels of PAI-1, an important molecule in the processes of fibrinolysis and a validated fibrinolysis biomarker, were lower in sepsis survivors. PAI-1 is considered an independent risk factor for sepsis-associated organ failure and death (53) and higher PAI-1 levels have been reported in sepsis non-survivors on days 1, 4, and 8 (54). Previous studies have associated lower PAI-1 levels with weight loss (55). While lower PAI-1 levels in our study may be related to the lower body weight of sepsis survivors, they may also suggest that the processes of fibrinolysis are impaired in mouse sepsis survivors. Collectively, these findings support the previously proposed concept of *persistent inflammation-immunosuppression and catabolic syndrome* in sepsis survivors (14).

We also found that increased peripheral inflammation and innate immune hyporesponsiveness coexist with brain alterations, including dysregulation in cholinergic signaling and increased neuroinflammation in mouse sepsis survivors. Basal forebrain cholinergic neurons have been implicated in the regulation of cognitive functions, including attention, learning, and memory, and motivation, emotional states, and cerebral blood flow (23). Neurodegeneration of these neurons is directly implicated in cognitive impairment in Alzheimer’s disease (23). Our results indicate a previously unrecognized vulnerability of basal forebrain cholinergic neurons among sepsis survivors, indicated by decreased numbers of ChAT-positive neuronal bodies in this area. Basal forebrain cholinergic neurons innervate the cortex (neocortex), hippocampus, and other forebrain regions. In sepsis survivors, we observed a trend toward higher AChE activity and significantly increased *Ache* gene expression in the cortex. In addition, AChE activity was significantly increased in the cortex and *Ache* gene expression was unaltered in the hippocampus. Considering the role of AChE as the main enzyme degrading acetylcholine, these observations may reflect depletion of acetylcholine in the synaptic cleft and cholinergic hypofunction. We also found region-specific alterations in the gene expression of *Chrm1* (coding for M1 mAChR), a decrease in the hippocampus and no difference in cortex. Given the important role of forebrain cholinergic signaling in cognition, our observations suggest that brain cholinergic dysfunction can be an important underlying event contributing to the cognitive deficits in sepsis survivors. Accordingly, enhancing brain cholinergic signaling might be useful for treating cognitive deterioration in sepsis survivors.

Neuroinflammation is a major component in brain functional dysregulation in sepsis-associated encephalopathy (9). Microglia activation has been directly associated with a systemic inflammatory reaction in a case–control study which focused on the distribution of immunophenotype of microglia in brain tissue collected from patients with sepsis (11). Neuronal degeneration in the cortex and hippocampus, in parallel with increased inflammation, and blood brain barrier disruption has been reported as early as 12–24 h after CLP surgery in mice (56). In this study, we observed morphological alterations in microglia found in the cortex, indicative of activation, and associated with upregulated *Iba1* gene expression. In line with the role of activated microglia as a major source of proinflammatory cytokines, we found increased *Il1b* and *Il6* gene expression in the cortex and hippocampus. This neuroinflammation is accompanied by dysregulated cholinergic signaling in these brain areas, indicative of cholinergic hypofunction. Previously, brain cholinergic signaling has been shown to control neuroinflammation; increased cholinergic signaling suppresses microglial activation, and the release of proinflammatory cytokines (57). Therefore, it is plausible that cholinergic hypofunction and uncontrolled microglial activation in the cortex and hippocampus contributes to increased neuroinflammation among sepsis survivors. These two findings raise the intriguing question of whether decreased brain cholinergic tone contributes to the focal brain inflammation. It is known that exacerbated peripheral and brain inflammatory activation and cytokine release have deleterious effects on neurons (16). In a case–control study systemic infection and inflammation was associated with microglia activation in brain tissue from patients who died of sepsis (11). We and others have demonstrated brain microglial activation in the presence of increased levels of peripheral serum cytokines (16, 33, 58). Our *in vitro* data shed additional light on the relationship between activated microglia and impaired cholinergic signaling in the cortex; microglial activation has a profound impact on cholinergic components, including ChAT and AChE, the main enzymes associated with acetylcholine synthesis and degradation, respectively. Accordingly, this loss of cholinergic anti-inflammatory control on brain inflammation may be at least partially caused by products released by activated microglia contributing (*via* currently unknown mechanism) to cholinergic neurodegeneration.

Brain cholinergic M1 mAChR signaling also regulates peripheral inflammation (17, 26, 29, 31). Cortex areas and the hippocampus innervated by cholinergic pathways interact, directly or through multisynaptic pathways, with the dorsal vagal complex, the brainstem integrative center of the inflammatory reflex, a vagus nerve-based powerful regulator of peripheral immune responses and inflammation (17, 52, 59, 60). Our findings highlight an interplay between systemic and brain inflammation, which can result in neuronal damage of brain cholinergic neurons and impaired cholinergic neurotransmission in murine sepsis survivors. These findings together with the previously indicated role of brain cholinergic signaling in cognition and the control of peripheral and local inflammation suggests that cholinergic dysfunction may further facilitate inflammation and this reciprocal relationship may be an important underlying event contributing to cognitive impairment in sepsis survivors. Accordingly, brain cholinergic dysfunction may be a relevant therapeutic target for alleviating the chronic illness observed among sepsis survivors.

## Ethics Statement

All animal experiments were performed in accordance with the National Institutes of Health Guidelines under protocols approved by the Institutional Animal Care and Use Committee (IACUC) and the Institutional Biosafety Committee (IBC) of the Feinstein Institute for Medical Research, Northwell Health, Manhasset, NY, USA.

## Author Contributions

VAP, NZ, MA, KJT, and BD designed research; NZ, MEA, HHS, HLP, SIV-F, KRA, KRL, MN, MEA, and VP performed research; NZ, MEA, HP, KA, KL, PO, MN, CNM, PW, MA, SSC, KJT, and VAP analyzed and interpreted data; NZ and VAP wrote the article; SIV-F, KRL, CNM, PW, MA, BD, and KJT provided additional comments and contributed to finalizing the article.

## Conflict of Interest Statement

The authors declare that the research was conducted in the absence of any commercial or financial relationships that could be construed as a potential conflict of interest.

## Supplementary Material

Figure S1 Choline acetyltransferase and DAPI immunostaining in basal forebrain (scale bar = 100 μm).

Figure S2 Cholinergic gene expression in cortex and hippocampus in sham-operated control mice **(A)** *Ache* gene expression. *P* = 0.022, Student’s *t-*test, *n* = 8 per group. **(B)** *Chrm1* gene expression. *P* = 0.0012, Student’s *t-*test, *n* = 8 per group.
