# Supplementary figures and images for: Forebrain Cholinergic Dysfunction and Systemic and Brain Inflammation in Murine Sepsis Survivors
Source: Front Immunol. 2017 Dec 15;8:1673. doi: 10.3389/fimmu.2017.01673 (PMC5736570; doi:10.3389/fimmu.2017.01673)

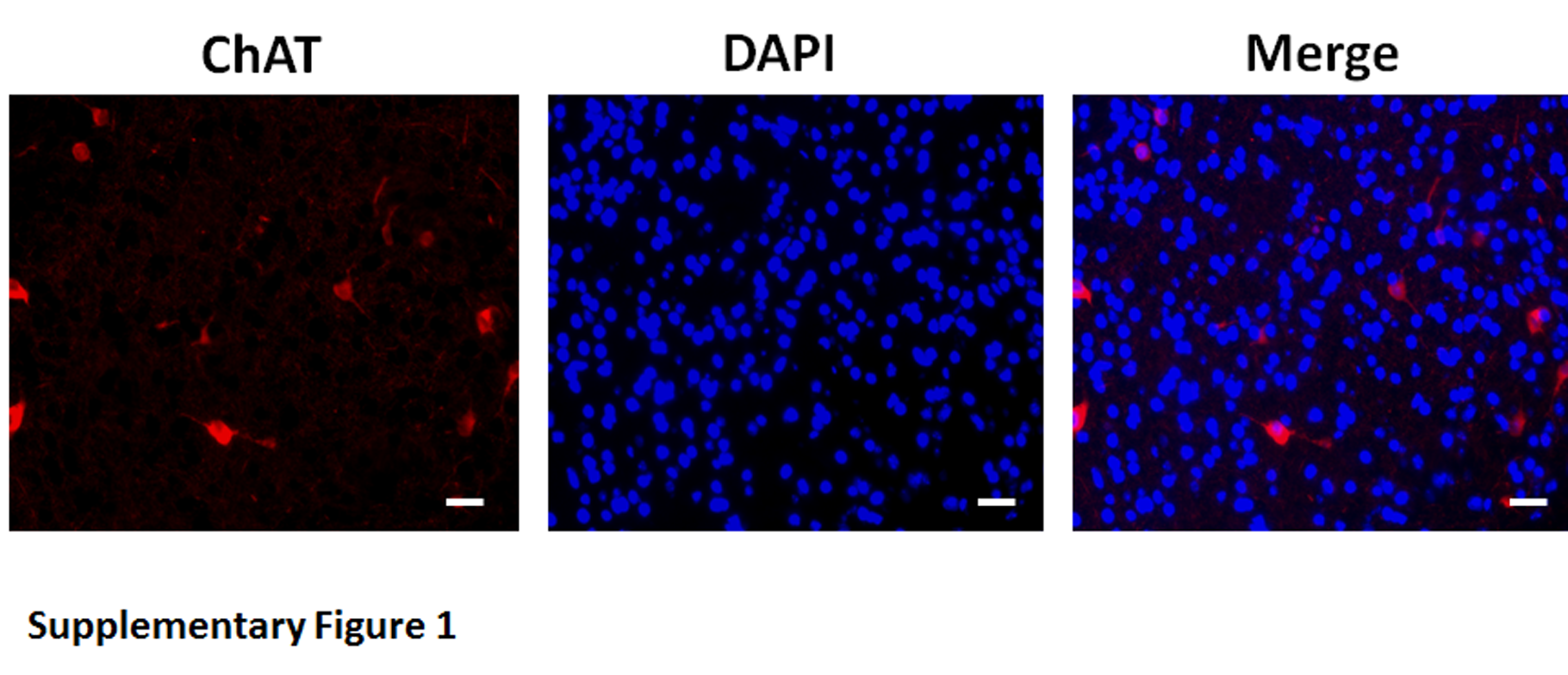

Supplement: Figure S1 — Choline acetyltransferase and DAPI immunostaining in basal forebrain (scale bar = 100 μm). [file Image_1.tif]

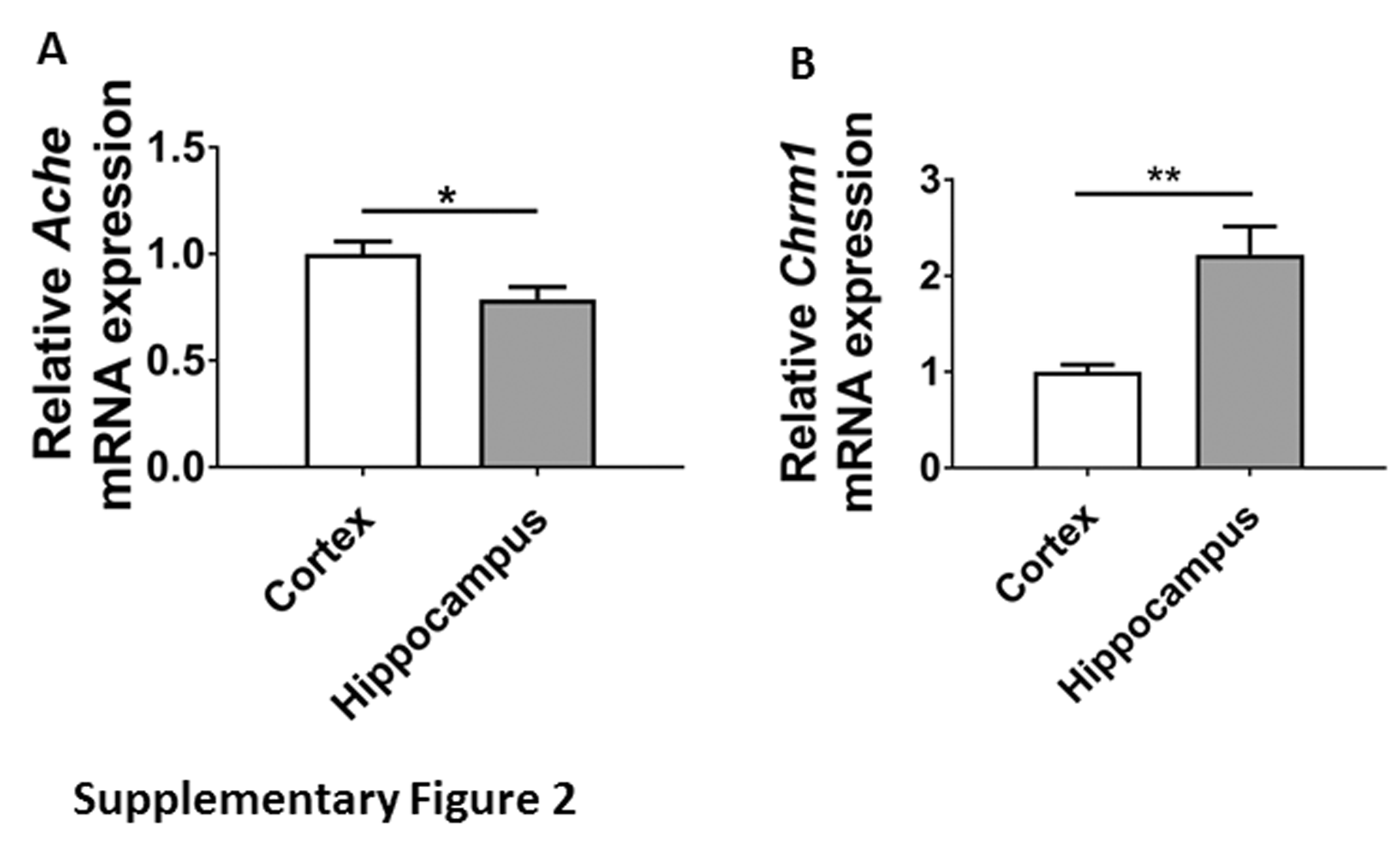

Supplement: Figure S2 — Cholinergic gene expression in cortex and hippocampus in sham-operated control mice (A) Ache gene expression. P = 0.022, Student’s t-test, n = 8 per group. (B) Chrm1 gene expression. P = 0.0012, Student’s t-test, n = 8 per group. [file Image_2.tif]
